# Non-Steroidal Anti-Inflammatory Drugs and Cancer Death in the Finnish Prostate Cancer Screening Trial

**DOI:** 10.1371/journal.pone.0153413

**Published:** 2016-04-21

**Authors:** Thea Veitonmäki, Teemu J. Murtola, Kirsi Talala, Kimmo Taari, Teuvo Tammela, Anssi Auvinen

**Affiliations:** 1 Department of Urology, Tampere University Hospital, Tampere, Finland; 2 School of Health Sciences, University of Tampere, Tampere, Finland; 3 School of Medicine, University of Tampere, Tampere, Finland; 4 Finnish Cancer Registry, Helsinki, Finland; 5 Department of Urology, University of Helsinki and Helsinki University Hospital, Helsinki, Finland; Innsbruck Medical University, AUSTRIA

## Abstract

Non-steroidal anti-inflammatory drugs (NSAIDs), especially aspirin, have been associated with lowered cancer incidence and mortality. We examined overall cancer mortality and mortality from specific cancer sites among the 80,144 men in the Finnish Prostate Cancer Screening Trial. Information on prescription drug use was acquired from the national drug reimbursement database. Over-the-counter use information was gathered by a questionnaire. Hazard ratios (HR) and 95% confidence intervals (CI) by prescription and over-the-counter NSAID use for overall and specific cancer deaths were calculated using Cox regression. During the median follow-up time of 15 years, 7,008 men died from cancer. Men with prescription NSAID use had elevated cancer mortality (HR 2.02 95% CI 1.91–2.15) compared to non-users. The mortality risk was increased for lung, colorectal and pancreas cancer mortality (HR 2.68, 95%CI 2.40–2.99, HR 1.91, 95% CI 1.57–2.32 and HR 1.93, 95% CI 1.58–2.37, respectively). The increased risk remained in competing risks regression (HR 1.11, 95% CI 1.05–1.18). When the usage during the last three years of follow-up was excluded, the effect was reversed (HR 0.69, 95% CI 0.65–0.73). Cancer mortality was not decreased for prescription or over-the-counter aspirin use. However, in the competing risk regression analysis combined prescription and over-the-counter aspirin use was associated with decreased overall cancer mortality (HR 0.76, 95% CI 0.70–0.82). Cancer mortality was increased for NSAID users. However, the risk disappeared when the last 3 years were excluded.

## Introduction

Epidemiologic studies have estimated that more than 20% of all human cancer cases are associated with chronic inflammation [1]. Inflammation has been thought to promote cancer by enhancing tumor cell proliferation and resistance to apoptosis. Inflammation also stimulates angiogenesis and tissue remodeling, which contributes to tumor cell invasion and progression [2–4].

The cyclooxygenase 2 enzyme (COX-2) is an inducible enzyme that facilitates inflammation by catalyzing the conversion of arachidonic acid to prostaglandins. COX-2 is commonly over-expressed in several cancers including esophageal, gastric, pancreatic, colorectal and prostate cancer [5–9].

Non-steroidal anti-inflammatory drugs (NSAIDs) that inhibit the COX-2 enzyme have been associated with lowered cancer incidence, progression and prolonged survival [10–15]. Especially in colorectal cancer, observational studies and clinical trials suggest that NSAIDs, particularly aspirin could prevent cancer development and progression [10]. Similarly, NSAID use has been linked to reduced risk of several other cancers [11–15]. Studies on NSAIDs on overall cancer mortality are sparse.

We examined cancer-specific and overall mortality by prescription and over-the-counter NSAID usage among the study population of the Finnish Prostate Cancer Screening Trial.

## Materials and Methods

### Study cohort

The Finnish Prostate Cancer Screening Trial (FinRSPC) is the largest component of the European Randomized Study of Prostate Cancer Screening (ERSPC) trial [16]. The protocol details have been described previously [17]. In short, between 1996–1999 men aged 55–67 years from Tampere and Helsinki residential areas were identified from the population register of Finland. 80,144 men were randomly assigned into the screening arm (31,866 men) or control arm with no intervention (48,278 men). After exclusion of prevalent prostate cancer cases no other prevalent cancers were excluded.

The official causes of death in 1996–2012 were obtained from Statistics Finland [18]. During 1996–2003 a cause-of-death committee evaluated causes of death among men who had been previously diagnosed with prostate cancer following a standard protocol (a predetermined decision algorithm and a flow diagram) based on anonymised medical records including laboratory and imaging results [19]. A death was assigned to prostate cancer if there was evidence of progressive prostate cancer, indicated by the presence of metastases from prostate cancer. The results showed the official causes of death to be highly accurate (kappa 0.95 compared with cause-of-death committee) [20]. In Finland, the circumstances are good for proper cause-of-death determination and death certification because of high autopsy rates. Also, cause-of-death determination and death certification practices are directed, supervised and partly carried out by medical examiners. Lahti et al concluded that Finnish death certificate form, death certification practices and cause of death validation procedure serves the coding of causes of death for mortality statistics appropriately and form a relevant reference background to evaluation of epidemiological studies on mortality [21, 22]. Death certificate information includes primary, immediate and contributory causes of death. We considered primary cause of death recorded as International Classification with Disease (ICD-10) codes with lung (C34), colorectal (C18), pancreatic (C25), gastric (C16), liver (C22) excluding bile duct cancer, renal (C64), non-Hodgkin lymphoma (C81), bladder (C67) and central nervous system cancer (C71 and C72).

The hospitalization registry (HILMO) maintained by the National Institutes for Health and Welfare covers all Finnish health care units and records discharge dates and diagnoses recorded for inpatient episodes as ICD-10 codes. The information on co-morbidities was gathered from HILMO.

Information on socioeconomic status such as level of education, income, marital status was obtained from statistics Finland the longitudinal census data. The information was available for 12,855 men in the Finnish prostate cancer screening trial.

### Information on medication use

The Social Insurance Institution (SII) of Finland is a governmental agency providing reimbursements for the cost of physician-prescribed drug purchases in outpatient settings. The reimbursement is available for all Finnish residents. The database records for each purchase the date, as well as type, amount and dose of the drug. Purchases of prescription-free drugs are not recorded, neither are medications used by hospital inpatients [23]. The NSAIDs available/licensed in Finland during the study period are listed in S1 Table. The entire study cohort was linked to the purchase database for individual-level information on physician-prescribed NSAID purchases during 1995–2009 using unique personal identification number assigned to each Finnish resident. The medication data was obtained for 78,615 men (98.1% of the screening trial population).

Men invited to the third screening round (years 2004–2007) were mailed a questionnaire on over-the-counter NSAID and aspirin use since 1990 with the screening invitation. The questionnaire included questions about frequency and dosage. Additional information such as height and weight for BMI- calculation was acquired. The response rate among the screened men was 92.6% (11,052 participants).

The study protocol was reviewed and approved by the Tampere and Helsinki University Hospital Ethics committees (tracking numbers R09159 and R10167). Permission to use cancer registry data was obtained from the Research and Development Center for Welfare and Health (STAKES, currently part of the National Institute of Health and Welfare). The Ministry of Social Affairs and Health gave the permission to seek the information on prostate cancer cases from the medical records in 1999 and the National Institute for Health and Welfare continued the permission in 2010. Under these permissions, information could be attained of the non-consenting men in the screening arm and men in the control arm of the study. The consenting men in the screening arm gave their written informed consent. Patient information has been anonymized and de-identified prior to analysis.

### Statistical analysis

#### Prescription use

Cox proportional hazards regression was used to estimate hazard ratios (HR) and 95% confidence intervals (CIs) for overall cancer death and separately for deaths from lung, colorectal, pancreatic, gastric, liver, renal, bladder and central nervous system cancers and non-Hodgkin lymphoma, by medication usage. The risk was analyzed separately for all users of prescription NSAIDs and separately for aspirin and coxib users. Non-users of any NSAIDs or non-users of the drug/drug group being analyzed were used as a reference group. For each man in the study population, from the follow-up started at the date of screening trial randomization and ended at the date of death, emigration or common closing date (December 31th 2012), whichever came first.

Cox regression model was adjusted for age and simultaneous use of other medications (drugs used for diabetes, hypercholesterolemia and hypertension). Additional analysis was performed with adjustment for socioeconomic status.

NSAID usage after randomization was analyzed as time-dependent variable, with usage status updated separately for each year after baseline. Men were categorized as non-users until the year of the first recorded NSAID purchase. The status changed into NSAID user, which was maintained for each year with recorded purchases. Men who discontinued NSAID usage during the follow-up were categorized as previous users.

NSAIDs might be used for pain relief in metastatic disease. As this protopathic bias usually occurs during the final years of life a separate lag-time analyses excluding NSAID or aspirin usage during the final year and three years of follow-up were performed. Incurable, palliative stage of cancer can last from weeks to years and there are no exact criteria to define the best time lag to be applied in the mortality analysis. In observational studies on NSAID use and cancer risk, the used lag time has been 1–2 years [24].

The amount of usage between different NSAIDs was standardized by dividing the yearly milligram amount of drug used with the average daily dose, termed Defined Daily Dose (DDD) listed by the World Health Organization (WHO ATC/DDD index database) [25]. Duration of use was calculated as years with recorded NSAID purchases. Intensity, i.e. dosage per year was calculated by dividing the total number of DDDs purchased with years of usage (number of DDDs/year).

Dose-dependency was evaluated by stratifying the users by cumulative amount, duration or intensity of usage. P-values for trend were calculated by adding the cumulative usage and quartiles as continuous variables into the Cox regression model.

Subgroup analyses were conducted by stratifying the men by age at randomization, baseline malignancy and medication usage (cholesterol-lowering, anti-diabetic and antihypertensive medication). Additional analysis was done stratifying by BMI and socioeconomic status.

Effect modification by underlying co-morbidities was evaluated using the updated Charlson Co-morbidity Score [26] described in S2 Table. The index was calculated based on ICD-10 codes from HILMO. We lacked the information on the severity of liver disease and could not separate mild, moderate and severe disease. Thus, for any liver disease a score of the minimum two points was given.

Because NSAID users might have more co-morbidity, a Fine-Grey competing risk analysis was conducted adjusting analysis for non-cancer mortality as a competing risk.

#### Over-the-counter use

Separate analysis were done for overall over-the-counter NSAID use (ibuprofen, ketoprofen, dexibuprofen combined), ibuprofen and aspirin alone, and combined over-the-counter and prescription use. Analyses for dosage and frequency were done separately for ibuprofen and aspirin use (tablets per day, days per month).

All Cox regression analyses were performed using IBM SPSS statistical software (version 20, Chicago, Illinois, USA). The Fine-Grey competing risk analysis was conducted by STATA software (Stata Corporation, College Station, Texas).

## Results

### Population characteristics

The median age at randomization was 59 years for both NSAID and aspirin users. Prevalence of prescription NSAIDs usage was 77.8% of the whole study population during 1995–2009. The prevalence of prescription aspirin usage was 9.1%.

During the median follow-up of 15 years, 7,008 cancer deaths occurred, 5,527 among NSAID prescription users and 1,481 among non-users. The most common cause of cancer death was lung cancer (1,561 deaths among NSAID users and 405 in non-users). There occurred 664 colorectal cancer deaths during the follow-up (518 deaths among NSAID users, 146 among non-users and 51 among prescription aspirin users) (Table 1).

**Table 1:** Population Characteristics of Prescription Non-steroidal Anti-Inflammatory drug users and non-users in the Finnish Prostate Cancer Screening Trial.

	Prescription NSAID use				Prescription aspirin use	
	Never		Ever		Ever	
*Characteristics of Participants*						
Number of participants	17,509	22.2%	61,318	77.8%	7,183	9.1%
Median Age	59.0 (55–67)		59.0 (55–67)		59.0 (55–67)	
Median BMI	25.64		26.47		27.13	
Baseline cancer diagnosis (any)	629	3.6%	2650	4.3%	326	4.5%
Charlson Co-morbidity index						
0	12,923	74.0%	42,085	68.6%	4,267	59.4%
1	796	4.5%	3,010	4.9%	642	8.9%
2 or greater	3,758	21.5%	16,043	26.2%	2,274	31.7%
*Cancer death*						
Overall cancer death	1,481	8.5%	5,527	9.0%	603	8.4%
Lung cancer death	405	2.3%	1,561	2.6%	188	2.6%
Colorectal cancer death	146	0.8%	518	0.8%	51	0.7%
Pancreatic cancer death	133	0.8%	491	0.8%	54	0.8%
Gastric cancer death	83	0.5%	207	0.3%	19	0.3%
Hepatic cancer	89	0.5%	291	0.5%	30	0.4%
Renal cancer	44	0.3%	201	0.3%	28	0.4%
Non-Hodgkin Lymphoma	52	0.3%	182	0.3%	25	0.3%
Bladder cancer	37	0.2%	127	0.2%	14	0.2%
Central nervous system cancer	51	0.3%	126	0.2%	12	0.2%
*Prevalence of medication use*	No of men	%	No	%	No	%
NSAID use						
-prescription usage^a^	-	-			6,092	84.8%
-self-reported over-the-counter(n of users/n of respondents)^b^	1,135/1,904	59.6%	6,635/9,148	72.5%	633/947	66.8%
Aspirin use						
-prescription usage ^a^	1,096	6.3%	6,117	10.0%	-	-
-self-reported over-the-counter use (n of users/n of respondents)^b^	842/2,067	40.7%	4,884/9,734	50.2%	530/947	55.9%
Anti-diabetic drugs ^c^	2,792	15.9%	13,133	21.4%	2,280	31.7%
Cholesterol-lowering drugs ^d^	5,223	29.8%	27,695	45.2%	5,414	75.4%
Antihypertensive drugs ^e^	9,769	55.8%	43,324	70.7%	6,573	91.5%

^a^ Information on physician-prescribed purchases reimbursed by the Social Insurance Institution (SII) of Finland between 1995 and prostate cancer diagnosis, death, or common closing date Dec 31, 2009, whichever comes first. Information obtained from comprehensive national prescription database

^b^ Self-reported, prescription-free use of non-steroidal anti-inflammatory drugs among the participants of the third screening round of the Finnish Prostate Cancer Screening Trial

^c^ Includes oral antidiabetic drugs (metformin, sulfonylureas, thiazilidinediones, dipeptidyl peptidase-4 inhibitors, meglitinides, α-glucosidase inhibitors and glugacon-like peptide agonists) and insulin

^d^ Includes statins, fibric acid derivatives, bile acid-binding resins and acipimox

^e^ Includes diuretics, beta-blockers, calcium-channel blockers, angiotensin-converting enzyme inhibitors and angiotensin receptor blockers

Compared to the NSAID non-users, aspirin users had more comorbidity and were more often users of antidiabetic, antihypertensive and cholesterol-lowering drugs (Table 1).

### Over-the-counter use and cancer mortality

Overall cancer mortality did not differ by over-the-counter NSAID usage, but colorectal cancer mortality was increased in users compared to non-users (HR 3.19, 95% CI 1.25–8.12). Nevertheless, colorectal cancer mortality risk was not associated with usage of the most common over-the-counter NSAID, ibuprofen (HR 0.99, 95% CI 0.36–2.77). No clear dose-dependency was observed (Table 2).

**Table 2:** Over-the-counter NSAID use and cancer mortality by amount and frequency of use in the Finnish Prostate Cancer Screening Trial during 1996–2012.

**NSAID**	n of death/men	Overall cancer death^a^		Lung cancer death		Colorectal cancer death		Pancreatic cancer death
		HR(95%CI)^b^		HR(95%CI)^b^		HR(95%CI)^b^		HR(95%CI)^b^
*Over-the-counter use*^*c*^								
no	925/28	ref	98	ref	4	ref	2	ref
yes(NSAID)		1.00(0.81–1.24)		0.96(0.64–1.44)		3.19(1.25–8.12)		0.83(0.45–1.51)
yes (ibuprofen)	10,876/407	1.43(0.94–2.10)	111	1.45(0.70–2.86)	43	1.02(0.37–2.86)	47	2.47(0.60–10.21)
*Frequency*								
*Days per month*								
-no use	1,381/45	ref	11	ref	6	ref	6	ref
-2 tabl or less	1,466/42	1.01(0.66–1.53)	14	1.45(0.66–3.20)	4	0.69(0.19–2.45)	5	0.97(0.30–3.20)
-over 2 tabl	1,127/36	1.07(0.69–1.66)	10	1.30(0.53–3.06)	4	0.87(0.25–3.10)	6	1.41(0.45–4.39)
*Amount*								
*Tablets per day*								
-no use	1,098/36	ref	10	ref	6	ref	4	ref
-1 and under	2,390/83	1.20(0.81–1.78)	22	1.21(0.57–2.60)	11	0.91(0.34–2.48)	8	1.13(0.34–3.75)
-over one	2,054/66	1.14(0.76–1.72)	21	1.39(0.65–2.96)	7	0.69(0.23–2.07)	11	1.87(0.59–5.88)
**Aspirin**	n of men/death	Overall cancer death^a^	n of deaths	Lung cancer death	n of deaths	Colorectal cancer death	n of deaths	Pancreatic cancer death
		HR(95%CI)^b^		HR(95%CI)^b^		HR(95%CI)^b^		HR(95%CI)^b^
*Over-the-counter use*^*c*^								
no	840/22	ref	6	ref	3	ref	1	ref
yes	10,961/413	1.60(1.04–2.45)	113	1.61(0.71–3.65)	44	1.28(0.40–4.12)	48	4.01(0.53–29.06)
*Frequency*								
*Days per month*								
-no use	1,151/38	ref	9	ref	7	ref	4	ref
-non daily use	1,554/48	1.02(0.67–1.56)	11	1.04(0.43–2.52)	6	0.67(0.22–1.99)	11	2.41(0.77–7.58)
-daily use	1,930/77	1.24(0.83–1.84)	19	1.27(0.57–2.83)	7	0.62(0.21–1.82)	6	0.85(0.24–3.05)
*Amount*								
*Tablets per day*								
-no use	996/30	ref	8	ref	6	ref	3	ref
-1 and under	3,565/129	1.27(0.85–1.90)	32	1.20(0.55–2.61)	13	0.64(0-24-1.71)	14	1.39(0.40–4.88)
-over 1	1,122/41	1.40(0.88–2.25)	11	1.50(0.60–3.74)	6	0.97(0.31–3.04)	7	2.65(0.68–10.27)

a Overall Cancer death (lung, pancreatic, colorectal, kidney, bladder, non-hodgkin lymphoma, central nervous system cancer)

b Hazard ratio for cancer death by NSAID prescription use adjusted for age, randomization group, use of cholesterol-lowering medication, antihypertensive medication, antidiabetic medication

c Self-reported, prescription-free use of non-steroidal anti-inflammatory drugs among the participants of the third screening round of the Finnish Prostate Cancer Screening Trial

Over-the-counter aspirin use was associated with elevated overall cancer mortality (HR 1.60, 95% CI 1.04–2.45). The risk estimates were non-significantly elevated also for lung, colorectal and pancreatic cancer death (Table 2).

### Prescription NSAID use and cancer mortality

The overall cancer mortality was elevated for both current and past prescription NSAID usage (HR 2.02, 95% CI 1.91–2.15 and HR 1.48,95% CI 1.37–1.57, respectively) (Table 3). The mortality was similarly elevated for lung, colorectal, pancreatic (S3 Table) and other cancer types (S3 Table). The risk of cancer death increased with cumulative amount and intensity of use (Table 3). When non-cancer mortality was included in the analysis as a competing risk, NSAID use was remained associated with cancer mortality (HR 1.11, 95% CI 1.05–1.18).

**Table 3:** Overall cancer mortality and lag time analyses by amount, duration and intensity of non-steroidal anti-inflammatory drugs in the Finnish Prostate Cancer Screening Trial during 1996–2012.

	All NSAIDs			Aspirin		
		1 year excluded	3 years excluded		1 year excluded	3 years excluded
NSAID use	HR(95%CI)^a^	HR(95%CI)^a^	HR(95%CI)^a^	HR(95%CI)^a^	HR(95%CI)^a^	HR(95%CI)^a^
Non-users	Ref	Ref	Ref	Ref	Ref	Ref
Users	2.02(1.91–2.15)	1.74(1.64–1.84)	0.85(0.80–0.90)	1.03(0.85–1.26)	1.32(1.11–1.57)	1.31(1.12–1.52)
Previous users	1.48(1.37–1.57)	1.13(1.06–1.20)	0.69(0.65–0.73)	1.50(1.32–1.69)	1.37(1.20–1.56)	1.04(0.91–1.19)
Cumulative quantity of medication use^b^						
DDD quartiles						
1	1.15(1.07–1.25)	1.07(0.99–1.16)	0.68(0.33–0.73)	2.19(1.46–3.29)	1.50(1.16–1.92)	1.13(0.88–1.44)
2	1.50(1.39–1.62)	1.28(1.19–1.38)	0.68(0.33–0.73)	1.55(1.15–2.10)	1.34(1.13–1.58)	1.34(1.13–1.58)
3	2.38(2.22–2.55)	1.72(1.60–1.85)	0.73(0.68–0.79)	1.60(1.09–2.35)	1.37(1.11–1.69)	1.37(1.11–1.69)
4	2.22(2.07–2.39)	1.66(1.54–1.79)	0.72(0.66–0.77)	1.62(1.12–2.34)	1.26(1.02–1.56)	1.26(1.02–1.56)
p for trend (by DDD)	<0.001	<0.001	<0.001	0.05	0.23	0.01
p for trend (by quartiles)	<0.001	<0.001	<0.001	0.94	0.53	<0.001
Duration of medication use^c^						
Year quartiles^d^						
1	1.59(1.48–1.70)	1.34(1.26–1.44)	0.76(0.71–0.81)	1.55(1.17–2.05)	1.30(1.11–1.52)	1.06(0.91–1.24)
2	1.84(1.73–1.97)	1.43(1.34–1.53)	0.73(0.68–0.77)	1.71(1.19–2.47)	1.49(1.22–1.83)	1.19(0.97–1.46)
3	1.77(1.62–1.93)	1.43(1.31–1.56)	0.63(0.57–0.69)	1.96(1.29–3.00)	1.25(0.96–1.64)	1.05(0.80–1.38)
4	1.67(1.52–1.84)	1.33(1.21–1.46)	0.52(0.47–0.57)	1.68(1.08–2.63)	1.38(1.08–1.77)	0.86(0.66–1.12)
p for trend (by year)	<0.001	<0.001	<0.001	0.02	0.43	<0.001
p for trend (by quartiles)	<0.001	<0.001	<0.001	0.56	0.33	<0.001
Intensity of medication use (DDDs/year)^d^						
Intensity quartile						
1	1.13(1.05–1.22)	1.08(1.00–1.16)	0.67(0.62–0.72)	2.19(1.55–3.09)	1.38(1.10–1.72)	1.11(0.90–1.38)
2	1.31(1.20–1.42)	1.14(1-05-1.24)	0.59(0.54–0.64)	1.76(1.27–2.42)	1.50(1.26–1.79)	1.11(0.93–1.34)
3	1.83(1.70–1.97)	1.41(1.31–1.52)	0.68(0.63–0.73)	1.23(0.78–1.94)	1.40(1.12–1.74)	0.95(0.75–1.19)
4	2.78(2.61–2.97)	2.00(1.87–2.14)	0.86(0.80–0.92)	1.54(1.09–2.18)	1.14(0.92–1.40)	1.02(0.83–1.25)
p for trend (by yearly dose)	<0.001	<0.001	0.02	0.13	0.91	0.02
p for trend (by quartiles)	<0.001	<0.001	<0.001	0.65	0.75	<0.001

^a^ Hazard ratios of cancer death from Cox regression analysis adjusted for age, use of cholesterol-lowering medication, antihypertensive medication, antidiabetic medication and the screening trial arm.

^b^ Estimated by including cumulative daily dose (DDD) quartiles for NSAID use after randomization: overall NSAID use 1–34 doses(1st quartile), 35–95 doses(2nd quartile), 96–275 doses(3rd quartile), over 275 doses(4^th^ quartile), aspirin 1–3.3 doses(1st quartile), 3.4–10 doses(2nd quartile), 10.1–22.5 doses(3rd quartile), 22.6 of more doses(4th quartile)

^c^ quartiles for duration of NSAID use after randomization: overall NSAID use 1 year(1st quartile), 2 years (2nd quartile), 3 years and 4 (3rd quartile) 5 or over years (4th quartile). Use on aspirin after randomization 1 year (1st quartile), 2 years (2nd quartile), 3 (3rd quartile), 4 or over years (4th quartile).

^d^ Quartile intensity cut-points: Overall NSAID use: 1–20 DDDs/year (1^st^ quartile), 21–35 DDDs/year (2^nd^ quartile), 36–67 DDDs/year (3^rd^ quartile) and 68 DDDs/year or more (4^th^ quartile); Aspirin use: 0.25–3 DDDs/year (1^st^ quartile), 3.25–5 DDDs/year (2^nd^ quartile), 5.25–7 DDDs/year (3^rd^ quartile), over 7 DDDs/year (4^th^ quartile)

Exclusion of NSAID usage during the final year of follow-up did not remove the association with elevated overall cancer mortality (HR 1.74, 95% CI 1.64–1.84), but exclusion of medication usage during the three final years of follow-up reversed the association, with a lowered mortality in NSAID users (HR 0.85, 95% CI 0.80–0.90) (Table 3). In the lagged analysis excluding the final three years, overall cancer mortality decreased with increasing duration of NSAID use, but no clear dose-dependence by amount or intensity of use was observed.

Overall cancer mortality was not elevated in prescription aspirin users, when including usage during the entire follow-up (Table 3). However, in the lagged analysis, ongoing aspirin use was associated with an increased risk of cancer death (HR 1.31 95% CI 1.12–1.52 in the analysis excluding aspirin use from the three final years). Furthermore, in the analysis for duration of use, the cancer mortality risk seems to decrease with duration of aspirin use (Table 3). When non-cancer mortality was included in the analysis as a competing risk, combined prescription and over-the-counter aspirin use was associated with decreased overall cancer mortality (HR 0.76, 95% CI 0.70–0.82).

Analyzed separately, the risk of death was elevated among NSAID users for most cancer types, except CNS cancers (S3 Table). Risk increase by aspirin usage was not significant for any specific cancer type, while coxib users had an increased risk only for renal cancer death (HR 1.95, 95% CI 1.13–3.37).

In lagged analysis excluding the final three years, lung, colorectal and pancreatic cancer mortality were reduced among NSAID users compared to non-users (S4 Table)

### Subgroup analysis

**Fig 1:**
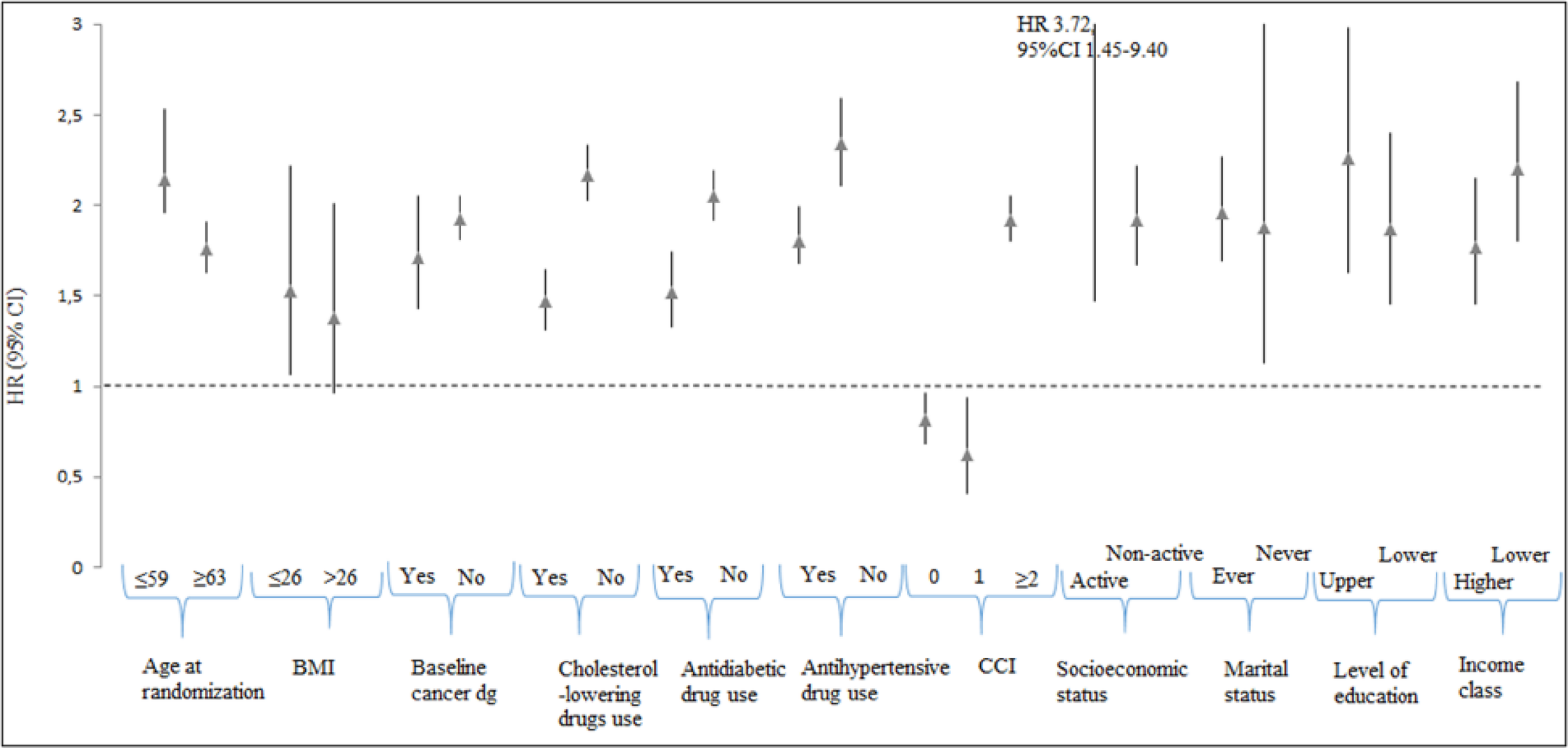
Overall cancer cancer mortality by NSAID current use versus non-use stratified by patient characteristics in the Finnish Prostate Cancer Screening Trial.

Cancer mortality remained elevated among NSAID users in almost all subgroups (Fig 1). In the analysis stratified by Charlson index (CCI), NSAID users with the least co-morbidities (CCI 0 or 1) had decreased overall cancer mortality compared with non-users (HR 0.81, 95% CI 0.68–0.96 and HR 0.62, 95% CI 0.41–0.94, respectively). The overall cancer mortality risk was significantly increased among the NSAID users with CCI>1 (HR 1.92, 95% CI 1.80–2.05) relative to non-users. Aspirin usage was not significantly associated with risk of cancer death in any subgroup with the exception of men with most co-morbidity (Charlson index 2 or more), in whom lowered risk was observed compared to non-users (S5 Table). NSAID and aspirin users with higher income had also an elevated cancer mortality risk despite the cancer type compared to non-users. Similar results were observed for pensioners (Fig 1 and S5 Table).

## Discussion

We found elevated cancer mortality among prescription NSAID and aspirin users. However, this was mostly explained by NSAID usage during the final years before death, as exclusion of medication use during the last three years of follow-up reversed the association to a protective level. In the analysis incorporating other causes of death as competing causes, the cancer mortality risk remained elevated for NSAID use. In the main analysis, cancer mortality was not decreased for prescription or over-the-counter aspirin use. However, in the competing risk regression aspirin use was associated with decreased overall cancer mortality. Our results show the critical importance of the timing of NSAID usage in relation to cancer death and are consistent with a protective effect of NSAIDs in general and aspirin

Previous observational studies and even clinical trials have suggested that NSAIDs, particularly aspirin could prevent cancer development and progression, especially in colon cancer. Most previous studies have shown reduced cancer mortality for aspirin and NSAID users. Information on NSAID use in majority of previous studies was obtained from surveys conducted years before cancer deaths, usually with no up-dated information on exposures during the final years of life. Therefore, the results from our lagged analysis are probably most comparable with previous studies, and show concordant results. Also, we observed decreased cancer mortality for men with less comorbidity (Charlson index 0 or 1). Elevated cancer mortality was observed in the subgroup of men with CCI over 1, demonstrating the confounding by comorbidity. However, when non-cancer mortality was adjusted as a competing risk, the overall cancer mortality risk remained elevated for NSAID use. Our study supports the previous findings by showing that decreased risk of cancer death not only pertains to some cancer types, but also overall risk of cancer death is decreased among NSAID users.

Our results on aspirin and cancer mortality differ from many previous studies. However, in the 3 years lag time analysis a decreasing trend for cancer mortality was observed for duration of use. A pooled analysis of randomized trials of daily aspirin for prevention of vascular events found a substantial reduction in overall cancer mortality during follow-up after 5 years on aspirin [27]. In our study, the median time for prescription aspirin use was only 2 years. Beneficial effects of aspirin may require a long exposure: in two large randomized trials aspirin needed to be used daily for more than 5 years with a 10-year latency period before the risk of colorectal cancer was reduced [28]. Furthermore, in the competing risk analysis aspirin use was associated with decreased overall cancer mortality. Thus, a possible protective association for aspirin use cannot be excluded. Some studies have reported similar findings to our study. In the National Health and Nutrition Examination Survey (NHANES I), adverse associations between aspirin use and bladder (RR 12.31, 95% CI 2.98–50.80) and brain cancer mortality (RR 3.13, 95% CI 1.09–9.00) were observed, but only for women [29]. In a large cohort study, aspirin use was not associated with pancreatic cancer mortality [30].

In our study, prescription aspirin was reimbursable only for secondary prevention of cerebrovascular disorders, which was shown by higher Charlson index and higher prevalence of usage of other drug groups in prescription aspirin users. Therefore, the prescription aspirin users in our study were not entirely comparable to previous studies where aspirin has mostly been used in primary prevention. Our information on over-the-counter aspirin use was collected only during the third screening round in 2004–2008 for a subset of the subjects. Therefore, we had limited ability to evaluate long-term over-the-counter usage.

In a recent study among colorectal cancer patients, users of prescription NSAIDs and pre-diagnostic prescription aspirin had a decreased survival as compared with non-users [31]. In a cohort study of colorectal cancer patients, overall mortality was slightly lower with aspirin use up to 5 years, whereas after 10 years there was an increase in mortality (HR 1.94, 95% CI.26-2.99). High-dose NSAID use was associated with increased mortality (HR 1.41, 95% CI 1.26–1.56) [32]. Pre-diagnostic use of aspirin or non-aspirin NSAIDs did not reduce lung cancer death and high use of ibuprofen was associated with an increased risk in the VITamins And Lifestyle (VITAL) study [33]. However, these survival studies may not be comparable with mortality studies.

The different results for aspirin and non-aspirin NSAIDs and cancer mortality in this study are most likely due to several factors. Firstly, indication of use is different for the drugs. Prescription aspirin was used in small dose combination (25mg) with dipyridamole for secondary prevention of vascular events. Prescription NSAIDs are used mainly for relief of pain and inflammatory symptoms and taken as needed. Secondly, the cancer preventive mechanism of aspirin has thought to be via the anti-platelet pathway. Circulating platelets play an important role in cancer progression and metastasis [34]. Aspirin inhibits platelet aggregation and it has been suggested that this could delay or even prevent cancer progression and metastasis. Traditionally, NSAIDs’ potential anti-cancer mechanisms are thought to work via the COX-2 pathway by reducing inflammation. Recently, also COX-2-independent pathways have been discovered [35].

The strengths of our study include a large population-based cohort, comprehensive and detailed individual-level data on prescription NSAID purchases. We did not have information about over-the-counter usage for the whole study cohort but we could address the effect for a smaller population of men attending the third screening round. Previous studies have not separated over the counter and prescription usage. We were also able to evaluate separately ongoing and discontinued use, and could control the impact of co-morbidities and other medications.

Our study has also some limitations. We did not have information on cancer characteristics such as histology, stage and grade that predict risk of cancer death. Also, we did not have the information on the time of diagnosis nor possible recurrence. However, the information was missing from NSAID users and non-users alike, thus it is unlikely to cause differential bias.

It has been estimated that 35% of all cancer deaths in 2001 were attributable to potentially modifiable risk factors such as smoking, alcohol use, obesity, physical inactivity, unsafe sex, low fruit and vegetable intake [36]. We lacked the information on these risk factors. These factors could have differed by NSAID usage and possibly affect cancer outcomes and cause confounding. The information on family history of cancer was also lacking, with the exception of prostate cancer. We had self-reported information on BMI only for a proportion of the study population. On the other hand, socioeconomic status is a proxy indicator for many life-style factors. In the separate subgroup analysis, the cancer mortality remained elevated among NSAID users compared to non-users after adjustment for socioeconomic status. There might, however, be some residual confounding by factors that were not adequately controlled by such proxy indicator. The population consisted only of Finnish predominantly Caucasian males. Therefore, our results may not be generalizable to women or to other ethnic groups.

Finally, our data on prescription NSAID usage was based on medication purchases, with no information on the actual consumption. There might be exposure misclassification because prescribed drugs may be used only partially or not at all. In contrast, actual NSAID usage might be underestimated as approximately 28% of all NSAID purchases in Finland are over-the-counter [37]. Aspirin is more likely to be used regularly for preventive purposes.

## Conclusion

We observed an elevated overall cancer, lung and pancreatic cancer mortality risk for prescription NSAID usage compared to non-usage. This was explained by NSAID usage during the final years of life, as exclusion of the last three years of usage diminished the risk increase to a protective effect. Prescription aspirin use was not associated with decreased cancer mortality. However, in the competing risk regression the mortality risk decreased for combined prescription and over-the-counter use. Our results support a protective effect of NSAIDs against cancer death. A possible preventive association for aspirin use cannot be excluded.

## Supporting Information

S1 Table NSAIDs used among the study population during 1996–2009. (DOCX)

S2 Table Updated Charlson co-morbidity index scoring system. (DOCX)

S3 Table Hazard ratios for cancer mortality by Non-steroidal anti-inflammatory drug users versus non-users among the 80, 144 men attending the Finnish prostate cancer screening trial. (DOCX)

S4 Table Lung, colorectal and pancreatic cancer mortality and 3 years excluded lag time analyses by amount, duration and intensity of non-steroidal anti-inflammatory drugs Finnish Prostate Cancer Screening Trial During 1996–2012. (DOCX)

S5 Table Overall cancer, lung cancer and colorectal cancer mortality by NSAID current use versus non-use stratified by patient characteristics in the Finnish Prostate Cancer Screening Trial. (DOCX)
