# Supplementary material for: Non-Steroidal Anti-Inflammatory Drugs and Cancer Death in the Finnish Prostate Cancer Screening Trial
Source: PLoS One. 2016 Apr 21;11(4):e0153413. doi: 10.1371/journal.pone.0153413 (PMC4839624; doi:10.1371/journal.pone.0153413)
Supplement: S1 Table — (DOCX) [file pone.0153413.s001.docx]

**S1 Table. NSAIDs available in Finland during 1996-2009.**

| **Prescription NSAID** | | **Over-the-counter NSAID** |
| --- | --- | --- |
| The entire study period | Part of the study period (years) |  |
| aspirin | aseclofenac since 1999 | aspirin |
| ibuprofen | meloxicam since 1996 | ibuprofen |
| ketoprofen | sulindac until 2007 | ketoprofen |
| tiaprofen | tolfenic acid until 2008 | dexibuprofen |
| diclofenac | tenoxicam until 2008 |  |
| indomethacin | lornoxicam 2002-06 |  |
| mefenamic acid | nimesulide 1999-2002 |  |
| nabumetone | dexibuprofen 2002-05 |  |
| acetaminophen | fenbutatson 1995-1998 |  |
|  | celecoxib since 2000 |  |
|  | rofecoxib 2000-2005 |  |
|  | etoricoxib since 2003 |  |
|  | valdecoxib 2003-2005 |  |
