# Supplementary material for: Non-Steroidal Anti-Inflammatory Drugs and Cancer Death in the Finnish Prostate Cancer Screening Trial
Source: PLoS One. 2016 Apr 21;11(4):e0153413. doi: 10.1371/journal.pone.0153413 (PMC4839624; doi:10.1371/journal.pone.0153413)
Supplement: S2 Table — (DOCX) [file pone.0153413.s002.docx]

**S2 Table. Updated Charlson co-morbidity index scoring system^a^**

| *Condition* | *Score* |
| --- | --- |
| Chronic pulmonary disease | 1 |
| Rheumatologic disease | 1 |
| Diabetes with end-organ damage | 1 |
| Renal disease | 1 |
|  |  |
| Hemiplegia | 2 |
| Dementia | 2 |
| Congestive heart failure | 2 |
| Mild liver disease | 2 |
| Any malignanacy including leukemia and lymphoma | 2 |
|  |  |
| AIDS/HIV positive | 4 |
| Moderate or severe liver disease | 4 |
|  |  |
| Metastatic tumour | 6 |
|  |  |
| Maximum score | 24 |

a Quan et al. Updating and validating the Charlson comorbidity index and score for risk adjustment in hospital discharge abstracts using data from 6 countries. Am J Epidemiol. 2011 Mar 15;173(6):676-82.
