# Supplementary material for: Non-Steroidal Anti-Inflammatory Drugs and Cancer Death in the Finnish Prostate Cancer Screening Trial
Source: PLoS One. 2016 Apr 21;11(4):e0153413. doi: 10.1371/journal.pone.0153413 (PMC4839624; doi:10.1371/journal.pone.0153413)
Supplement: S3 Table — (DOCX) [file pone.0153413.s003.docx]

**S3 Table. Hazard ratios for cancer mortality by Non-steroidal anti-inflammatory drug users versus non-users among the 80, 144 men attending the Finnish prostate cancer screening trial**

| Cancer death | NSAID | | Aspirin | | Coxibs | |
| --- | --- | --- | --- | --- | --- | --- |
|  | HR (95% CI)^a^ | HR (95% CI)^b^ | HR (95% CI)^a^ | HR (95% CI)^b^ | HR (95% CI)^a^ | HR (95% CI)^b^ |
| *Lung cancer* |  |  |  |  |  |  |
| User | 2.27(2.03-2.53) | 2.68(2.40-2.99) | 1.12(0.82-1.53) | 1.49(1.09-2.05) | 0.83(0.63-1.10) | 0.92(0.70-1.22) |
| Previous user | 1.40(1.25-1.57) | 1.56(1.39-1.75) | 1.35(1.08-1.68) | 1.78(1.42-2.22) | 1.00(0.84-1.18) | 1.11(0.94-1.32) |
|  |  |  |  |  |  |  |
| *Colorectal Cancer* |  |  |  |  |  |  |
| User | 1.64(1.35-2.00) | 1.91(1.57-2.32) | 0.41(0.17-0.98) | 0.57(0.23-1.37) | 1.06(0.69-1.63) | 1.16(0.75-1.78) |
| Previous user | 1.42(1.17-1.72) | 1.47(1.30-1.90) | 1.15(0.77-1.72) | 1.59(1.06-2.40) | 1.03(0.77-1.38) | 1.14(0.85-1.53) |
|  |  |  |  |  |  |  |
| *Pancreatic* |  |  |  |  |  |  |
| User | 1.79(1.46-2.18) | 1.93(1.58-2.37) | 0.72(0.36-1.45) | 0.87(0.43-1.73) | 1.67(1.15-2.42) | 1.76(1.21-2.56) |
| Previous user | 1.41(1.15-1.71) | 1.48(1.21-1.81) | 1.08(0.70-1.67) | 1.27(0.82-1.99) | 1.61(1.25-2.08) | 1.72(1.33-2.22) |
|  |  |  |  |  |  |  |
| *Gastric cancer* |  |  |  |  |  |  |
| User | 1.22(0.90-1.66) | 1.41(1.03-1.92) | 0.56(0.18-1.74) | 0.74(0.24-2.33) | 1.09(0.58-2.06) | 1.19(0.63-2.24) |
| previous user | 1.29(0.97-1.71) | 1.42(1.07-1.89) | 1.05(0.56-1.97) | 1.38(0.73-2.63) | 1.27(0.85-1.90) | 1.40(0.94-2.10) |
|  |  |  |  |  |  |  |
| *Hepatic* |  |  |  |  |  |  |
| User | 1.32(1.02-1.72) | 1.38(1.06-1.86) | 0.86(0.38-1.93) | 1.06(0.47-2.40) | 1.07(0.61-1.86) | 1.11(0.64-1.94) |
| Previous user | 1.11(0.86-1.42) | 1.14(0.88-1.46) | 1.12(0.66-1.92) | 1.33(0.77-2.28) | 1.02(0.71-1.48) | 1.08(0.74-1.56) |
|  |  |  |  |  |  |  |
| *Non-hodgkin* |  |  |  |  |  |  |
| User | 1.74(1.26-2.34) | 1.97(1.43-2.71) | 0.90(0.33-2.43) | 1.14(0.42-3.09) | 0.82(0.36-1.85) | 0.88(0.49-1.99) |
| Previous user | 1.18(0.85-1.65) | 1.29(0.92-1.79) | 1.05(0.52-2.13) | 1.30(0.64-2.67) | 0.88(0.52-1.48) | 0.96(0.57-1.62) |
|  |  |  |  |  |  |  |
| *Renal* |  |  |  |  |  |  |
| User | 2.25(1.65-3.08) | 2.39(1.74-3.82) | 0.69(0.22-2.15) | 0.80(0.26-2.51) | 1.88(1.09-3.26) | 1.95(1.13-3.37) |
| Previous user | 1.51(1.09-2.10) | 1.88(1.14-2.19) | 1.67(0.95-2.93) | 1.91(1.08-3.38) | 1.07(0.65-1.76) | 1.12(0.68-1.83) |
|  |  |  |  |  |  |  |
| *Bladder* |  |  |  |  |  |  |
| User | 2.50(1.76-3.78) | 2.62(1.78-3.86) | 1.32(0.49-3.57) | 1.64(0.60-4.47) | 1.25(0.55-2.85) | 1.22(0.53-2.77) |
| Previous user | 1.58(1.06-2.40) | 1.60(1.07-2.40) | 1.16(0.49-2.50) | 1.35(0.59-3.099 | 1.55(0.95-2.55) | 1.54(0.93-2.52) |
|  |  |  |  |  |  |  |
| *CNS* |  |  |  |  |  |  |
| User | 0.76(0.49-1.18) | 0.89(0.57-1.39) | 1.33(0.49-3.56) | 1.80(0.70-5.18) | 0.18(0.03-1.27) | 0.20(0.03-1.40) |
| Previous user | 1.35(0.96-1.91) | 1.51(1.07-2.13) | 1.14(0.50-2.58) | 1.64(0.72-3.77) | 1.24(0.72-2.15) | 1.38(0.80-2.39) |

a Age adjusted hazard ratios of cancer mortality from Cox regression analysis.

b Hazard ratios of cancer mortality from Cox regression analysis adjusted for age, use of cholesterol-lowering medication, antihypertensive medication, antidiabetic medication and the screening trial arm.
