# Supplementary material for: Non-Steroidal Anti-Inflammatory Drugs and Cancer Death in the Finnish Prostate Cancer Screening Trial
Source: PLoS One. 2016 Apr 21;11(4):e0153413. doi: 10.1371/journal.pone.0153413 (PMC4839624; doi:10.1371/journal.pone.0153413)
Supplement: S4 Table — (DOCX) [file pone.0153413.s004.docx]

**S4 Table. Lung, colorectal and pancreatic cancer mortality and 3 years excluded lag time analyses by amount, duration and intensity of non-steroidal anti-inflammatory drugs Finnish Prostate Cancer Screening Trial During 1996-2012.**

|  | Lung cancer mortality  3 years excluded | Colorectal Cancer mortality  3 years excluded mortality | Pancreatic cancer mortality  3 years excluded |
| --- | --- | --- | --- |
|  | HR(95%CI)^a^ | HR(95%CI)^a^ | HR(95%CI)^a^ |
| non-user | ref | ref | ref |
| user | 0.87(0.79-0.97) | 0.82(0.67-1.00) | 0.74(0.60-0.91) |
| previous user | 0.65(0.58-0.73) | 0.72(0.59-0.86) | 0.64(0.52-0.77) |
|  |  |  |  |
| Cumulative quantity of medication use^b^ | |  |  |
| DDD quartiles |  |  |  |
| 1 | 0.67(0.60-0.77) | 0.72(0.56-0.89) | 0.57(0.45-0.72) |
| 2 | 0.66(0.57-0.76) | 0.79(0.63-0.99) | 0.63(0.49-0.80) |
| 3 | 0.69(0.60-0.80) | 0.76(0.59-0.97) | 0.64(0.49-0.82) |
| 4 | 0.82(0.71-0.95) | 0.48(0.36-0.66) | 0.67(0.52-0.87) |
| p for trend | 0.000 | 0.000 | 0.000 |
|  |  |  |  |
| Duration of medication use^c^ |  |  |  |
| Year quartiles^d^ |  |  |  |
| 1 | 0.76(0.67-0.86) | 0.82(0.67-1.01) | 0.63(0.51-0.79) |
| 2 | 0.72(0.63-0.81) | 0.73(0.60-0.91) | 0.62(0.49-0.77) |
| 3 | 0.65(0.53-0.78) | 0.62(0.46-0.85) | 0.63(0.47-0.85) |
| 4 | 0.56(0.46-0.67) | 0.34(0.23-0.51) | 0.57(0.03-0.49) |
| p for trend | 0.000 | 0.000 | 0.000 |
|  |  |  |  |
| Intensity of medication use (DDDs/year)^d^ | |  |  |
| Intensity quartile |  |  |  |
| 1 | 0.62(0.54-0.71) | 0.82(0.67-1.02) | 0.60(0.48-0.76) |
| 2 | 0.62(0.53-0.73) | 0.52(0.35-0.69) | 0.53(0.40-0.70) |
| 3 | 0.65(0.56-0.76) | 0.69(0.54-0.89) | 0.66(0.52-0.85) |
| 4 | 0.95(0.84-1.08) | 0.70(0.55-0.90) | 0.68(0.53-0.87) |
| p for trend | 0.000 | 0.000 | 0.000 |

a Hazard ratios of cancer death from Cox regression analysis adjusted for age, use of cholesterol-lowering medication, antihypertensive medication, antidiabetic medication and the screening trial arm.

b Estimated by including cumulative daily dose (DDD) quartiles for NSAID use: overall NSAID use 1-34 doses(1st quartile), 35-91 doses(2nd quartile), 92-265 doses(3rd quartile), over 265 doses(4^th^ quartile

c quartiles for duration of NSAID use: overall NSAID use 1 year(1st quartile), over 1 to 3 years (2nd quartile), over 3 to 5 years (3rd quartile) and over 5 years (4th quartile).

d Quartile cut-points: Overall NSAID use: 2-20 DDDs/year (1^st^ quartile), 21-34 DDDs/year (2^nd^ quartile), 35-67 DDDs/year (3^rd^ quartile) and 68 DDDs/year or more (4^th^ quartile)
