# Supplementary material for: Non-Steroidal Anti-Inflammatory Drugs and Cancer Death in the Finnish Prostate Cancer Screening Trial
Source: PLoS One. 2016 Apr 21;11(4):e0153413. doi: 10.1371/journal.pone.0153413 (PMC4839624; doi:10.1371/journal.pone.0153413)
Supplement: S5 Table — (DOCX) [file pone.0153413.s005.docx]

**S5 Table. Overall cancer, lung cancer and colorectal cancer mortality by NSAID current use versus non-use stratified by patient characteristics in the Finnish Prostate Cancer Screening Trial.**

|  | Overall cancer death | | Lung cancer | | Colorectal | |
| --- | --- | --- | --- | --- | --- | --- |
|  | NSAID | Aspirin | NSAID | Aspirin | NSAID | Aspirin |
|  | HR (95% CI)^a^ | HR (95% CI)^a^ | HR (95% CI)^a^ | HR (95% CI) ^a^ | HR (95% CI)^a^ | HR (95% CI)^a^ |
| *Patient characteristics* |  |  |  |  |  |  |
| Age at randomization |  |  |  |  |  |  |
| 55 or 59 years | 2.14(1.96-2.53) | 1.30(0.95-1.77) | 2.41(2.05-2.83) | 1.09(0.58-2.05) | 2.57(1.93-3.40) | 0.73(0.18-2.94) |
| 63 or 67 years | 1.76(1.63-1.91) | 0.90(0.70-1.56) | 2.54(2.19-2.95) | 1.67(1.16-2.41) | 1.32(1.00-1.74) | 0.48(0.15-1.51) |
| P for interaction | 0.23 | 0.22 | 0.87 | 0.88 | 0.72 | 0.72 |
| BMI^b^ |  |  |  |  |  |  |
| above median | 1.53(1.06-2.22) | 0.32(0.04-2.27) | 1.81(0.77-4.26) | - | 1.06(0.34-3.28) | 1.43(0.19-10.99) |
| below median | 1.38(0.96-2.01) | 1.39(0.51-3.79) | 1.03(0.54-1.96) | 0.86(0.19-6.27) | 3.10(1.06-9.12) | - |
| P for interaction | 0.04 | 0.01 | 0.28 | 0.29 | 0.70 | 0.15 |
| Baseline cancer diagnosis^c^ |  |  |  |  |  |  |
| yes | 1.71(1.43-2.05) | 0.85(0.42-1.72) | 2.54(1.74-3.72) | 1.41(0.45-4.47) | 2.93(1.72-4.97) | - |
| no | 1.93(1.81-2.05) | 1.05(0.85-1.28) | 2.45(1.32-2.02) | 1.47(1.06-2.04) | 1.64(1.32-2.02) | 0.62(0.26-1.51) |
| P for interaction | 0.04 | 0.05 | <0.001 | <0.001 | 0.74 | 0.64 |
| Medication use |  |  |  |  |  |  |
| Cholesterol-lowering drugs^d^ |  |  |  |  |  |  |
| Yes | 1.47(1.31-1.64) | 1.00(0.78-1.29) | 1.87(1.52-2.29) | 1.46(0.98-2.19) | 1.52(.03-2.23) | 0.93(0.38-2.26) |
| No | 2.17(2.03-2.33) | 1.02(0.75-1.40) | 2.83(2.48-3.22) | 1.48(0.89-2.47) | 1.92(1.53-2.41) | - |
| P for interaction | 0.81 | 0.82 | 0.50 | 0.55 | 0.20 | 0.20 |
| Antidiabetic drug use^e^ |  |  |  |  |  |  |
| Yes | 1.52(1.33-1.74) | 0.70(0.46-1.05) | 2.06(1.54-2.75) | 1.03(0.49-2.19) | 1.82(1.15-2.80) | 0.38(0.05-2.75) |
| No | 2.05(1.92-2.19) | 1.19(0.95-1.48) | 2.56(2.28-2.89) | 1.62(1.15-2.30) | 1.80(1.45-2.24) | 0.64(0.24-1.71) |
| P for interaction | 0.22 | 0.22 | 0.09 | 0.09 | 0.42 | 0.42 |
| Antihypertensive drug use^f^ |  |  |  |  |  |  |
| Yes | 1.80(1.68-1.99) | 1.03(0.84-1.27) | 2.30(2.18-2.87) | 1.54(1.10-2.18) | 1.57(1.23-2.01) | 0.65(0.27-1.57) |
| No | 2.34(2.11-2.59) | 1.12(0.62-2.02) | 2.58(2.15-3.10) | 1.23(0.46-3.11) | 2.39(1.72-3.31) | - |
| P for interaction | 0.61 | 0.61 | 0.50 | 0.51 | 0.92 | 0.92 |
|  |  |  |  |  |  |  |
| Charlson co-morbidity index^g^ |  |  |  |  |  |  |
| 0 | 0.81(0.68-0.96) | 0.99(0.58-1.68) | 0.80(0.58-1.10) | 0.95(0.45-2.02) | 0.37(0.15-0.94) | - |
| 1 | 0.62(0.41-0.94) | 0.31(0.08-1.26) | 0.71(0.36-1.40) | 0.47(0.06-3.40) | 0.55(0.05-5.629 | - |
| 2 or greater | 1.92(1.80-2.05) | 0.96(0.73-1.11) | 2.70(2.48-3.06) | 1.36(0.97-1.91) | 1.70(1.38-2.09) | 0.52(0.22-1.27) |
| P for interaction | 0.05 | 0.05 | 0.49 | 0.50 | 0.88 | 0.88 |
| Socioeconomic status^h^ |  |  |  |  |  |  |
| economically non-active | 1.92(1.67-2.22) | 1.53(1.06-2.21) | 2.86(2.21-3.72) | 2.28(1.30-4.00) | 2.09(1.28-3.39) | - |
| economically active | 3.72(1.47-9.40) | 1.75(0.40-7.62) | 1.79(0.38-8.36) | - | - | - |
| p for interaction | 0.85 | 0.86 | 1.00 | 1.00 | 1.00 | 1.00 |
| Marital status^i^ |  |  |  |  |  |  |
| never married | 1.88(1.13-3.13) | 0.94(0.23-3.89) | 3.78(1.44-9.89) | 3.36(0.76-14.76) | 10.29(1.16-92.41) | - |
| ever married | 1.96(1.69-2.27) | 1.61(1.11-2.32) | 2.76(2.11-3.60) | 1.99(1.08-3.66) | 2.00(1.22-3.31) | - |
| p for interaction | 0.65 | 0.64 | 0.79 | 0.79 | 0.88 | 0.88 |
| Level of education^j^ |  |  |  |  |  |  |
| upper level of education | 2.26(1.63-2.98) | 0.66(0.21-2.07) | 4.02(2.14-7.54) | 0.79(0.11-5.79) | 3.49(1.35-9.02) | - |
| lower level of education | 1.87(1.45-2.40) | 1.98(1.15-3.40) | 2.55(1.65-3.94) | 4.01(1.92-8.42) | 4.32(1.70-10.96) | - |
| p for interaction | 0.60 | 0.63 | 1.00 | 1.00 | 1.00 | 1.00 |
| Income class^k^ |  |  |  |  |  |  |
| lower | 2.20(1.80-2.68) | 1.35(0.79-2.31) | 3.07(2.14-4.39) | 1.35(0.49-3.66) | 1.60(0.75-3.41) | - |
| higher | 1.77(1.45-2.15) | 1.94(1.22-3.00) | 2.60(1.83-3.70) | 2.85(1.44-5.65) | 2.91(1.57-5.40) | - |
| p for interaction | 0.34 | 0.31 | 1.00 | 1.00 | 1.00 | 1.00 |

a Hazard ratios of cancer mortality from Cox regression analysis adjusted for age, use of cholesterol-lowering medication, antihypertensive medication, antidiabetic medication and the screening trial arm.

b Based on self-reported information from the participants of the third screening round of the Finnish Prostate Cancer Screening Trial, cutpoint of median BMI 26.30.

c Any cancer diagnosis at the time of randomization

d Includes statins, fibric acid derivatives, bile acid-binding resins, ezetimibe and acipimox

e Includes oral antidiabetic drugs (metformin, sulfonylureas, thiazilidinediones, dipeptidyl peptidase-4 inhibitors, meglitinides, α-glucosidase inhibitors and glugacon-like peptide agonists) and insulin

f Includes diuretics, beta-blockers, calcium-channel blockers, angiotensin-converting enzyme inhibitors and angiotensin receptor blockers

g Charlson co-morbidity index see S2 Table

h Socioeconomic status economically active (employed or self-employed), non-active (unemployed, retired, student)

i Marital status. Ever married (married or registered partnership, divorced or widow). Never married (single)

j Level of education divided into two groups: upper secondary level education (or lower) and lowest level tertiary education (or higher)

k Income class cut point of 30,000 euros per year
